# Effect of Vicia sativa L. on Motility, Mortality and Expression Levels of hsp Genes in J2 Stage of Meloidogyne hapla

**DOI:** 10.2478/jofnem-2023-0009

**Published:** 2023-04-14

**Authors:** Renata Dobosz, Łukasz Flis, Jan Bocianowski, Tadeusz Malewski

**Affiliations:** Institute of Plant Protection-National Research Institute, Department of Entomology and Animal Pests, Węgorka 20, 60-318 Poznan, Poland.; Museum and Institute of Zoology, Polish Academy of Sciences, Wilcza 64, 00-679 Warsaw, Poland.; Department of Mathematical and Statistical Methods, Poznan University of Life Sciences, Wojska Polskiego 28, 60-637 Poznan, Poland.

**Keywords:** behavior, *hsp* genes, Meloidogyne hapla, motility, mortality, *Vicia sativa* seeds

## Abstract

Assuming that the seeds of *Vicia sativa* L. have a stressful effect on J2 stage *Meloidogyne hapla*, we undertook research on the effect of these seeds on the motility and mortality of J2 and determined the expression levels of selected *hsp* genes in J2. The assessment of the effect of *V. sativa* seeds on the motility of *M. hapla* specimens consisted of observing the movement of J2 immersed in a seed diffusate or in a tomato root filtrate at temperatures of 10, 17, and 21°C. In J2 treated with *V. sativa* (cv. Ina) seed diffusates, the expression level of *hsp* genes was determined by qPCR. J2 exposed to *V. sativa* diffusates were found to lose their motility, while their mortality did not exceed 30%. J2 in the seed diffusate were characterized by an increase in the expression levels of the *Mh-hsp*90, *Mh-hsp*1, and *Mh-hsp*43 genes. It is suggested that the *hsp*90 gene may be a potential bioindicator of the environmental impact on *Meloidogyne* nematodes. The impaired ability to move in J2 of *M. hapla* is attributable to the occurrence of *V. sativa* seeds in their habitat. These studies may contribute to developing methods of reducing crop damage caused by *M. hapla*.

The root-knot nematode *Meloidogyne hapla* Chitwood, 1949 represents a group of about 100 species of nematodes - obligate plant parasites Moens et al., 2009. This nematode thrives on over 100 species of dicotyledons and is a serious pest of numerous valued crops, mainly in temperate regions Wesemael et al., 2011; Elling, 2013.

The occurrence of the root-knot nematode is evidenced by the characteristic root swellings (galls) which are caused by the interplay between nematodes and the plant in the parasite-host interaction. The root galls are the location where female nematodes dwell with their adjacent egg masses containing eggs and juveniles of the second stage (J2), with the invasive J2 being noted for their ability to move and infect root tissues Moens et al., 2009.

After leaving the egg masses and entering the soil, invasive J2 are exposed to the external environment, its abiotic factors (i.e., humidity, pH, and temperature), and biotic elements (e.g., fungi or plants) Wu et al., 2018; Zhou et al., 2018; Wang et al., 2019; Hua et al., 2020. The optimal ranges of abiotic factors and the availability of the host plant roots are conducive to the expansion of J2 into the root tissues. On entering the root tissues, J2 specimens become sedentary and undergo subsequent stages of individual development until they reach sexual maturity. In bisexual species, mature males leave the roots and die, having fertilized the females. Sedentary females lay their eggs into the egg masses attached to their bodies. The completion of one or several nematode developmental cycles during the growing season of the host plants may contribute to the growth of the root-knot nematode population in the soil or to maintaining it at a level close to the initial one Viaene and Abawi, 1996; Meressa et al., 2016; Dobosz and Krawczyk, 2019.

Common vetch *Vicia sativa* L.) is one of the plant species that contribute to the abundance of the northern root-knot nematode in the soil at a level close to the initial level pre-sowing. It is an annual plant grown across many regions of the world; in Poland, it is mainly grown in the autumn. Common vetch cultivation protects the soil against moisture loss and enriches it with nitrogen and organic matter. *V. sativa* also provides seeds and green forage for livestock Huang et al., 2017; Nguyen et al., 2020; Rodrigo-Comino et al., 2020; Grela et al., 2021. Plant-parasitic nematodes inhabiting the soil are known to be affected by common vetch. Observations of the development of the northern root-knot nematode in the soil under vetch cultivation showed a slight increase in the density of the nematode population and only sporadic root galling Dobosz and Krawczyk, 2019. The obtained results indicate that compared to other Fabaceae species studied, e.g. garden pea *Pisum sativum* L.) or faba bean *Vicia faba* L.), common vetch does not constitute the most suitable host for this nematode species.

Common vetch did not support the development of *Meloidogyne arenaria* (Neal, 1898) (race 2) and *Heterodera glicynes* Ichinoche, 1952 (race 4) Mosjidis et al., 1994. In addition to the limiting effect of common vetch on the development of the northern root-knot nematode population, its negative impact on other vegetation was also observed. In laboratory tests, a decrease in the germination capacity and growth rate was observed in seedlings of mungbean *Vigna radiata* L.) and mashbean *Vigna mungo* L.) cultivars exposed to extracts obtained from the overground parts of common vetch plants Zohaib et al., 2017. The observed results can be attributed to the presence of cyanogenic compounds found in the tissues and seeds of common vetch Ressler, 1972; Pitz et al., 1981; Megias et al., 2014). As a result of the hydrolysis of cyanogenic compounds, hydrogen cyanide (HCN) is released, which, inter alia, acts as a plant defense mechanism against herbivores Siegień and Bogatek, 2006; Zuk et al., 2020. HCN works by inhibiting cellular respiration by causing oxidative stress in animals Jones et al., 2000; Gillet et al., 2017.

As a result of oxidative stress or other stress factors (biotic or abiotic), metabolic changes occur in the cells of the body. The body’s response to stress stimuli encompasses, inter alia, the synthesis in the cells of specific proteins referred to as the so-called heat shock proteins (Hsps), also called chaperones Feder and Hofmann, 1999. Based on their molecular weight, Hsp proteins have been classified into six major families: Hsp110, Hsp90, Hsp70, Hsp60, Hsp40, and small heat shock proteins (sHsp), coded for by the corresponding heat shock *hsp* genes Gething and Sambrook, 1992; Feder and Hofmann, 1999; Kampinga et al., 2009; Carra et al., 2017. Depending on their expression level, Hsp proteins have a positive (chaperone function) or negative effect on the growth, development rate, reproduction, and life span of organisms Sørensen et al., 2003; Manière et al., 2014; Burnaevskiy et al., 2019; Wu et al., 2019b. The Hsp are essential for maintaining cellular homeostasis via promoting cell survival and preventing apoptotic processes in various cell types Rafiee et al., 2006. They protect cells from different stresses and play a key role in preventing damaged proteins from misfolding and refolding Samali and Orrenius, 1998. Numerous studies indicate that Hsp proteins and *hsp* genes play a significant role in the process of adaptation to the environment and survival of nematodes therein Seybold, 2015; Zhao and Wang, 2012; Him et al., 2009; Weinstein et al., 2019; Fanelli et al., 2021. De Luca et al. (2009) showed that in the plant-parasitic *Meloidogyne artiellia* Franklin, 1961, the *hsp*90 gene may play a crucial role in chemoreception, especially in the interaction between plant-parasitic nematodes and the root of the host plant.

For obligate plant parasites, which include root-knot nematodes, plants become available at the time of root formation, at the earliest in Phase 09 according to the BBCH scale (widely used in the European Union to identify phytophenological growth stages of particular crops, with the abbreviation BBCH standing for the German expression “Biologische Bundesanstalt, Bundessortenamt und CHemische Industrie”) Hack et al., 1992. Consequently, at this point, J2 stage specimens present in the soil have the opportunity to invade the roots. The vast majority of experiments focus on these observations. However, the scenario of J2 present in the soil being affected by plant seeds in early stages of germination (00-03 BBCH) cannot be ruled out.

The results of the authors’ studies as well as the aforementioned research findings led us to undertake research on the effects of *V. sativa* seeds on *M. hapla* J2 selected life activities. The aim of this study was twofold: 1) to determine the effect of common vetch seeds of cultivars Ina and Jaga on the motility and mortality in J2 stage *M. hapla*, and 2) to determine the cellular-level response patterns in the J2 stage when affected by common vetch (cv. Ina) seeds, by estimating the expression levels of selected *hsp* genes.

## Materials and Methods

*Plants and nematodes used for research*: Common vetch *Vicia sativa* L.) seeds were obtained from Plant Breeding DANKO, based in Szelejewo, Poland, and tomato *Solanum lycopersicum* L., cv. Betalux) seeds were obtained from from PNOS, based in Ożarów Mazowiecki, Poland. The seeds of Ina cultivar with the content of 7.9 mg% dry weight of cyanogenic compounds and the Jaga cultivar with the content of 3.5 mg% dry weight were used for the tests (COBORU, 2022).

*M. hapla* population was collected from a carrot *Daucus carota* L.) field and morphologically and genetically identified in accordance with Karssen’s (1999) and Petersen and Vrain’s (1996) diagnostic protocols, respectively. The population was maintained on tomato plants grown in a greenhouse under conditions of 20 ± 1°C and day and night length (16/8). *M. hapla* J2 juveniles were obtained from the eggs using the modified Baermann method described by Rodríguez-Kábana and Pope (1981). Only J2s that had left their eggshells within 24 hours prior to the start of the experiment were used in the study.

### Effect of *V. sativa* seeds on motility and mortality of J2 stage

*Surface sterilization of V. sativa seeds*: The seeds were surface-sterilized by succesive immersion in 70% ethanol and in 7.3% sodium hypochlorite solution for 20 minutes each Sobczak et al., 2005. Then, the seeds were washed vigorously in distilled water for 30 minutes. Seeds prepared in this manner were used to make diffusates.

*Preparation of tomato root diffusates*: Tomato *Solanum lycopersicum* L.) root diffusates were produced using the method elaborated by Devine et al. (1996). For this purpose, twenty tomato plants were grown in separate pots for four weeks. After this time, the pots were placed in funnels on funnel stands, which made it possible to collect the water which permeated the substrate, and to obtain the so-called soil filtrate. Then, the substrate with tomato roots was moistened by spraying its surface with 10 ml of distilled water. An additional 100 ml of distilled water was added to each pot. Excess water seeped through the substrate containing tomato roots and flowed out into a dish placed under the funnel. The first 50 ml of the filtrate collected from each pot, called tomato root diffusate, was mixed and used in the study of the motility and mortality of the J2 stage.

*Preparation of common vetch seed diffusates*: Eight types of diffusates were produced for the studies: two diffusates from surface-sterilized vetch seeds cultivars Ina and Jaga in water (Ina/Jaga surface-sterilized seeds + H_2_O), two diffusates from non-sterilized seeds of both vetch cultivars in water (Ina/Jaga seeds + H_2_O), two diffusates from surface-sterilized seeds of both vetch cultivars in the soil filtrate containing root secretions of tomato plants (Ina/Jaga surface-sterilized seeds + tomato root diffusates), and two diffusates from non-sterilized seeds of both vetch cultivars in the soil filtrate containing root secretions (Ina/Jaga seeds + root diffuses).

In order to prepare the diffusate from surface-sterilized common vetch seeds, 1 gram of previously surface-sterilized *V. sativa* seeds (cv. Ina and cv. Jaga) was weighed and each of the weighed seed portions was immersed for 24 hours in 100 ml of distilled water. The diffusate from non-sterilized seeds of both common vetch cultivars (Ina and Jaga) was prepared in a similar manner, but with the use of non-sterilized seeds instead. Both the diffusate from surface-sterilized common vetch seeds, as well as the diffusate from common vetch seeds not subjected to surface sterilization in the soil filtrate, were produced in the same manner, except that instead of 100 ml of distilled water, 100 ml of soil filtrate was used.

*Study of the effect of diffusates on the motility of the J2 stage*: Eight Petri dishes with a diameter of 3 cm were filled with 3 ml of each of the eight diffusates previously prepared, and 30 J2 specimens of *M. hapla* were transferred into each Petri dish. The dishes were placed at temperatures of 10, 17, and 21 ± 1°C. The control in the experiment were J2 stage specimens incubated in water and in soil filtrate. The experiment was performed twice, in six runs for each combination.

The nematodes were observed under a stereoscopic microscope 24 and 48 hours after exposure to seed diffusates. After this time, motile specimens and those that remained stationary after being touched with an entomological needle were counted Argentieri et al., 2008. The stationary specimens were placed in dishes with distilled water and observed again after 2 h and 24 h.

*J2 mortality assessment*: For mortality assessment of J2, stationary specimens were treated with 1 N NaOH according to the method by Chen and Dicson (2000). The percentage of stationary and dead specimens was determined using Abbott’s formula i=100×(1−nt/nc), in which i=% nematode immobility; nt=number of active nematodes after the treatment; and nc= number of active nematodes in the control (Argientieri et al., 2008).

*Statistical analysis*: The normality of the distribution of the four (immobile J2 after 24 h, immobile J2 after 48 h, immobile J2 immersed in water and immobile J2 after NaOH treatment) observed traits was tested using the Shapiro-Wilk normality test Shapiro and Wilk, 1965. The homogeneity of variance was tested using Bartlett’s test. Box’s M test tested multivariate normality and homogeneity of variance-covariance matrices. Non-normal traits were transformed using the power (Box-Cox) transformation with the lambda (λ) parameter at interval from −2 to 2 Kozak et al., 2008. Having the variables transformed and normally distributed, it was assumed that the data followed the multivariate normal distribution. A three-way (temperature, cultivar, treatment) multivariate analysis of variance (MANOVA) was performed. Next, a three-way analysis of variance (ANOVA) was carried out to determine the effects of temperature, cultivar, treatment and all interactions on the variability of all four observed traits. The mean values and standard deviations of traits were calculated. The Fisher’s least significant differences (LSDs) were calculated for individual traits, and on this basis, homogeneous groups were determined. The differences between combinations of the analyzed temperatures, cultivars and variants were verified by cluster analysis using the nearest neighbour method and Euclidean distances and presented as a dendrogram. The results were also analyzed using multivariate methods. A canonical variance analysis (CVA) was applied to present a multi-trait assessment of the similarity of the tested combinations of temperatures, cultivars, and variants in a lower number of dimensions with the least possible loss of information. Mahalanobis distance was suggested as a measure of “polytrait” combinations’ similarity Seidler-Łożykowska and Bocianowski, 2012, the significance of which was verified by means of critical value Dα,called “the least significant distance” Mahalanobis, 1936. Mahalanobis distances were calculated for all combinations. The relationships between observed traits were assessed on the basis of Pearson’s correlation. Relationships of four observed traits were depicted in a heatmap. The GenStat v. 18 statistical software package (VSN International) was used for the analyses.

## Effect of *V. sativa* seeds on the expression levels of *hsp* genes in J2 stage

*Exposure of J2 stage to V. sativa seed diffusates*: Only the diffusate from non-sterilized vetch seeds of the Ina cultivar in water was used in the study. The Ina cultivar is characterized by more than twice the content of cyanogenic compounds per gram of dry seeds compared to the Jaga cultivar. Three separate experiments were performed. They were carried out at a temperature of 21 ± 1°C. Each experiment was repeated three times. 200 specimens of the J2 stage of *M. hapla* were used for each run. In the first experiment, the nematodes were placed in Petri dishes in a diffusate for 24 hours. Each of the three Petri dishes with the diffusate contained 200 specimens of the J2 stage specimens. After this time, the immobile (paralysed) nematodes were transferred to Eppendorf tubes and immediately preserved with phenosol (RNA preservative reagent by A&A Biotechnology RNA) and frozen at -80°C, until obtaining the isolation of total RNA. In the second experiment, nematodes (200 specimens x 3 repetitions) were placed in a common vetch seed diffusate for 24 h and then transferred to water for 24 h. In this experiment, 24 hours after being transferred from the diffusate to water, almost all the nematodes regained the ability to move (approximately 8% of the specimens remained stationary in each run of the experiment). The specimens were then preserved with phenosol and immediately frozen at -80°C until RNA isolation. In the third experiment, J2 specimens (200 specimens × 3 repetitions) were left in the water for 24 hours, the control sample. The control sample was then preserved and frozen at -80°C.

*RNA extraction*: The isolation of total RNA from the three experiments described above was performed by the modified phenol-chloroform method with the use of the A&A Biotechnology kit Chomczynski and Sacchi, 1987. RNA extraction was performed in the pre-PCR room using disposable DNase- and RNase-free pipette tips with filters and test tubes. All stages of the experiment were performed at a temperature of approx. 4°C. The obtained RNA was stored at -80°C until further analysis. The quantity and quality of the obtained RNA was determined using the NanoDrop 1000 Spectrophotometer vs. 3.7 (Thermo Scientific). RNA integrity was assessed by electrophoresis using the RiboRuler High Range RNA Ladder (Thermo Scientific).

*cDNA synthesis*: DNase digestion of the obtained RNA and first-strand cDNA synthesis were performed with the iScript ™ gDNA Clear cDNA Synthesis Kit (Bio-Rad, Hercules, CA, USA) according to the procedure described by the manufacturer. The DNAse reaction mixture, in the amount of 0.5 μl and 1.5 μl of DNAse buffer for each 14 μl of RNA, was used to treat the residual DNA in the isolated RNA at a concentration of 10 ng / μl. The mixture thus prepared was incubated for 5 min at 25°C. It was then incubated for 5 min at 75°C to inactivate DNase and transferred to ice. The RNA prepared in this manner was used for the synthesis of cDNA. However, if longer storage was necessary, the RNA sample was placed at -80°C.

For cDNA synthesis, we used 16 μl of DNase treated RNA and 4 μl of the reaction mixture containing i.a. iScript reverse transcriptase enzyme, RNAase inhibitor, primers oligo (dT), free nucleotides (dNTPs), magnesium chloride (MgCl_2_ and polymerase stabilizing substances. The synthesis was carried out in the following steps: priming – 5 min at 25°C; reverse transcription (cDNA synthesis) – 20 min at 46°C; inactivation of the enzyme reverse transcriptase – 1 min at 95°C. The obtained first strand of cDNA was used directly in a *quantitative polymerase chain reaction* (qPCR) analysis or stored at -20°C.

*Primer design*: The primers for the tested genes *Mh*–*hsp*90, *Mh*–*hsp*1, *Mh*–*hsp*60, *Mh*–*hsp*43 and *Mh*–*hsp*12.3 used in the qPCR reaction were designed using the PRIMER3 vs. 0.4.0 program Untergasser et al., 2012. The Merck company performed the synthesis of the designed primers. The reference malate dehydrogenase *Mdh* gene and primers for amplifying a fragment of this gene (forward 5´–GAAAGCCAGGGATGACAC–3´, reverse 5´–AGAAAAGCATTGGGACAG–3´) were selected on the basis of research by Wu et al. (2019a). The *Mdh* gene has been shown to be one of the most suitable reference genes for gene expression studies in *M. hapla.*

*qPCR reaction*: We analyzed the expression of five *hsp* genes *Mh*–*hsp*90, *Mh*–*hsp*1, *Mh*–*hsp*60, *Mh*–*hsp*43, and *Mh*–*hsp*12.3). Expression of the 2^ΔΔ^ Ct method Livak and Schmittgen, 2001 was used in calculating the relative ratio, but instead of the value being 2, the correct amplification efficiency was used. We used a noise-resistant iterative nonlinear regression algorithm (qPCR miner) to determine the efficiency of the PCR reaction Zhao and Fernald, 2005. The expression levels are indicated as the fold-change normalized to the control (untreated diffusate), normalized to the value of 1.

qPCRs were performed on the 7500 qPCR system (Applied Biosystems, Waltham, MA, USA). Reactions were carried out using SsoAdvanced Universal SYBR Green Supermix kit (Bio-Rad), while cycle threshold (Ct) estimates were obtained using the relative quantification module in the software package. PCR reactions were performed in a final volume of 20 μl containing 1.5 μl of the cDNA sample, 3.0 μl of the primer mix (5 micromoles of each primer), 10 μl of the 2x SsoAdvanced Universal SYBR Green Supermix, and 5.5 μl of H_2_O. The assay included a no template control and each of the test cDNAs from three biological replications. According to the instructions for this kit, after 30 sec. at 95°C, the cycling conditions were as follows: 40 cycles at 95°C for 15 sec. and 59°C for 60 sec. To validate the specificity of amplification, a post-amplification melt-curve analysis was performed. Amplicons were first denatured at 95°C for 60 sec. and then cooled to 72°C, and the temperature was then gradually raised to 95°C. Fluorescence data were recorded continuously during this period, and analyzed subsequently using the Tm calling module in the SDS Software v1.4 software.

*Statistical analysis: Hsp* gene expression values were expressed as the mean fold difference. Statistically significant differences between treated and control samples (p≤0.01 based on *t*-Student test, using an online tool “Do my qPCR calculation”) are shown Tournayre et al., 2019.

## Results

### Effect of *V. sativa* seeds on motility and mortality of J2 stage

The results of the MANOVA performed indicated that all factors [temperature (T): Wilk’s λ=0.2932; *F*=83.19; cultivar (C): Wilk’s λ=0.3015; *F*=227.60; variant (V): Wilk’s λ=0.0705; *F*=79.86] and all interactions [T×C: Wilk’s λ=0.7850; *F*=12.64; T×V: Wilk’s λ=0.0894; *F*=33.22; C×V: Wilk’s λ=0.2618; *F*=32.48; T×C×V: Wilk’s λ=0.2568; *F*=16.09] were significantly different *P*<0.0001) with regard to all of the four quantitative traits. ANOVA indicated that the main effects of temperature, cultivar and variate, as well as all interactions, were significant for all the traits of study Table 1. The mean values and standard deviations for the observed traits indicated a high variability among the tested combinations, for which significant differences were found in terms of all the analyzed traits Table 2.

**Table 1:** Mean squares [%^2^] from three-way analysis of variance for observed traits.

Source of variation	d.f.	Immobile J2 after 24 h	Immobile J2 after 48 h	Immobile J2 immersed in water	Immobile J2 after NaOH treatment
Temperature (T)	2	8723.15***	38277.8***	943.39***	938.374***
Cultivar (C)	1	40703.81***	8983.6***	257.202***	262.371***
Variant (V)	5	9691.42***	34681.6***	583.498***	582.31***
TC	2	159.67*	3219.1***	47.557**	47.016**
TV	10	3228.36***	7990.8***	440.458***	441.152***
CV	5	5546.58***	2276.7***	184.671***	183.297***
TCV	10	2071.8***	884.7***	104.192***	104.979***
Residual	396	45.1	120.1	7.319	7.377

*P<0.05; **P<0.01; ***P<0.001; d.f. – the number of degrees of freedom.

After 48 hours, the specimens immersed in the tomato root diffusate and in water retained their motility. Differences in the effect of seed diffusates of the examined common vetch cultivars on the motility of J2 forms were observed. Ina cultivar seed diffusates (with a high content of cyanogenic compounds) influenced the behaviour of J2 stage through limiting their ability to move and caused paralysis at each of the tested temperatures, regardless of whether they were subjected to the sterilization process Table 2.

In the case of the Jaga cultivar (low content of cyanogenic compounds), J2 specimens became immobilized (paralyzed) when placed in seed diffusates prepared in the tomato root filtrate after 48 hours of immersion at 17°C and at 21°C, regardless of the duration of exposure. A complete loss of motility was observed in J2 forms of the nematode exposed to the diffusates from sterile common vetch (cv. Ina), prepared in tomato root filtrate, incubated at 21°C. When subjected to the diffusates of common vetch (cv. Jaga) seeds, 100% of J2 juveniles lost the ability to move after 48 hours of incubation at 21°C Table 2.

After being transferred into Petri dishes with water, J2 regained the ability to move (the paralysis subsided) at a rate of 90%. The mortality rate was 29% at the maximum, with the highest rate observed in the experiment variant using sterile seeds of the Ina cultivar, at a temperature of 21°C Table 2.

**Table 2:** Mobility of *Meloidogyne hapla* second-stage juveniles (J2) after exposed to water and *Vicia sativa* seed diffusates, and mortality after NaOH treatment. In table we presented meand values [In %] and standard deviations - s.d. [In %].

Combination	Temperature	Cultivar	Variant	Immobile J2 after 24 h	Immobile J2 after 48 h	Immobile J2 immersed in water	Immobile J2 after NaOH treatment
				**Mean ± s.d**.	**Mean ± s.d**.	**Mean ± s.d**.	**Mean ± s.d**.
1	10°C	Ina	J2+H_2_O	0 ± 0 i	0 ± 0 k	0 ± 0g	0 ± 0 f
2	10°C	Ina	J2+S+H_2_O	34.72 ± 13.062 de	29.44 ± 12.936 hi	2.778 ± 1.297 ef	2.778 ± 1.297 de
3	10°C	Ina	J2+StS+H_2_O	43.33 ± 12.713 c	30.56 ± 15.031 ghi	1.111 ±2.171 fg	1.111 ±2.171 ef
4	10°C	Ina	J2+RD	0 ± 0 i	0 ± 0 k	0±0g	0 ± 0 f
5	10°C	Ina	J2+S+RD	8.06 ± 4.597 h	22.22 ± 13.731 ij	1.389 ± 1.716 fg	1.667 ±2.247 ef
6	10°C	Ina	J2+StS+RD	20 ± 9.535 f	34.44 ±8.165 fgh	1.944 ± 1.716 fg	1.944 ± 1.716 ef
7	10°C	Jaga	J2+H_2_O	0 ± 0 i	0 ± 0 k	0±0g	0 ± 0 f
8	10°C	Jaga	J2+S+H_2_O	0 ± 0 i	0 ± 0 k	0±0g	0 ± 0 f
9	10°C	Jaga	J2+StS+H_2_O	0 ± 0 i	0 ± 0 k	0±0g	0 ± 0 f
10	10°C	Jaga	J2+RD	0 ± 0 i	0 ± 0 k	0±0g	0 ± 0 f
11	10°C	Jaga	J2+S+RD	0 ± 0 i	0 ± 0 k	0±0g	0 ± 0 f
12	10°C	Jaga	J2+StS+RD	0 ± 0 i	0 ± 0 k	0±0g	0 ± 0 f
13	17°C	Ina	J2+H_2_O	0 ± 0 i	0 ± 0 k	0±0g	0 ± 0 f
14	17°C	Ina	J2+S+H_2_O	18.33 ± 10.2 fg	14.72 ± 19.667 j	0±0g	0 ± 0 f
15	17°C	Ina	J2+StS+H_2_O	21.94 ± 15.6 f	24.72 ± 15.793 i	4.167 ±4.949 de	4.167 ± 4.949 d
16	17°C	Ina	J2+RD	0 ± 0 i	0 ± 0 k	0±0g	0 ± 0 f
17	17°C	Ina	J2+S+RD	36.39 ± 10.489 d	48.89 ± 15.33 d	0±0g	0 ± 0 f
18	17°C	Ina	J2+StS+ RD	36.11 ± 9.83 d	38.89 ± 14.094 efg	1.667 ±1.741 fg	1.667 ± 1.741 ef
19	17°C	Jaga	J2+ H_2_O	0 ± 0 i	0 ± 0 k	0±0g	0 ± 0 f
20	17°C	Jaga	J2+S+H_2_O	0 ± 0 i	0 ± 0 k	0±0g	0 ± 0 f
21	17°C	Jaga	J2+StS+H_2_O	0 ± 0 i	0 ± 0 k	0±0g	0 ± 0 f
22	17°C	Jaga	J2+RD	0 ± 0 i	0 ± 0 k	0±0g	0 ± 0 f
23	17°C	Jaga	J2+S+ RD	0 ± 0 i	45.83 ± 14.848 de	1.667 ±2.247 fg	1.667 ±2.247 ef
24	17°C	Jaga	J2+StS+RD	0 ± 0 i	39.17 ± 14.293 efg	0.556 ± 1.297 g	0.556 ± 1.297 f
25	21 °C	Ina	J2+H_2_O	0 ± 0 i	0 ± 0 k	0±0g	0 ± 0 f
26	21°C	Ina	J2+S+H_2_0	17.78 ± 10.184 fg	39.72 ± 11.322 ef	7.778 ±4.103 c	7.778 ±4.103 c
27	21°C	Ina	J2+StS+H_2_O	29.44 ± 9.727 e	38.33 ± 9.924 efg	29.167 ± 10.648 a	29.167 ± 10.648 a
28	21°C	Ina	J2+RD	0 ± 0 i	0 ± 0 k	0±0g	0 ± 0 f
29	21°C	Ina	J2+S+RD	53.61 ± 14.387 b	71.11 ± 22.398 c	1.389 ± 1.716 fg	1.389 ± 1.716 ef
30	21°C	Ina	J2+StS+RD	100 ± 0 a	100 ± 0 a	0.556 ± 1.297 g	0.556 ± 1.297 f
31	21°C	Jaga	J2+H_2_O	0 ± 0 i	0 ± 0 k	0±0g	0 ± 0 f
32	21°C	Jaga	J2+S+H_2_O	14.44 ± 7.566 g	44.17 ± 15.707 de	6.944 ±3.001 cd	6.944 ± 3.001 c
33	21°C	Jaga	J2+StS+H_2_O	16.67 ±6.816 fg	15.56 ± 7.698 j	10.556 ± 7.083 b	10.556 ± 7.083 b
34	21°C	Jaga	J2+RD	0 ± 0 i	0 ± 0 k	0 ± 0 g	0 ± 0 f
35	21°C	Jaga	J2+S+RD	20.83 ± 6.376 f	100 ± 0 a	2.778 ±3.978 ef	2.778 ± 3.978 de
36	21°C	Jaga	J2+StS+RD	18.33 ± 8.704 fg	84.17 ± 24.168 b	1.667 ± 2.659 fg	1.667 ±2.659 ef
LSD0.05				5.39	8.795	2.171	2.18

*J2+H_2_0 - juveniles of second stage immersed in water; J2+S+H_2_O - juveniles of second stage Immersed in seeds diffusates of *Vicia sativa* In water; J2+StS+H_2_O - juveniles of second stage immersed in surface-sterilized seeds diffusates of *Vicia sativa* in water; J2+RD - juveniles of second stage Immersed in root diffusates of S. *iycopersicum-*, J2+S+RD - juveniles of second stage immersed in seeds diffusates of *Vicia sativa* In root diffusates of S. *iycopersicum-*, J2+StS+RD - juveniles of second stage immersed in surface-sterilized seeds diffusates of *Vicia sativa* in root diffusates of S. *iycopersicum.* Means followed by the same letter are not statistically different.

The correlation analysis performed indicated statistically significant correlation coefficients between immobile J2 after 24 h and immobile J2 after 48 h *r*=0.7419), as well as immobile J2 immersed in water and immobile J2 after NaOH treatment *r*=1.000) Table 3.

**Table 3:** Correlation coefficients between observed traits.

Trait	Immobile J2 after 24 h	Immobile J2 after 48 h	Immobile J2 immersed in water	Immobile J2 after NaOH treatment
Immobile J2 after 24 h	1			
Iimmobile J2 after 48 h	0.7419***	1		
Immobile J2 immersed in water	0.2069	0.2241	1	
Immobile treatment J2 after NaOH	0.2066	0.2241	1***	1

***P<0.001.

**Figure 1:**
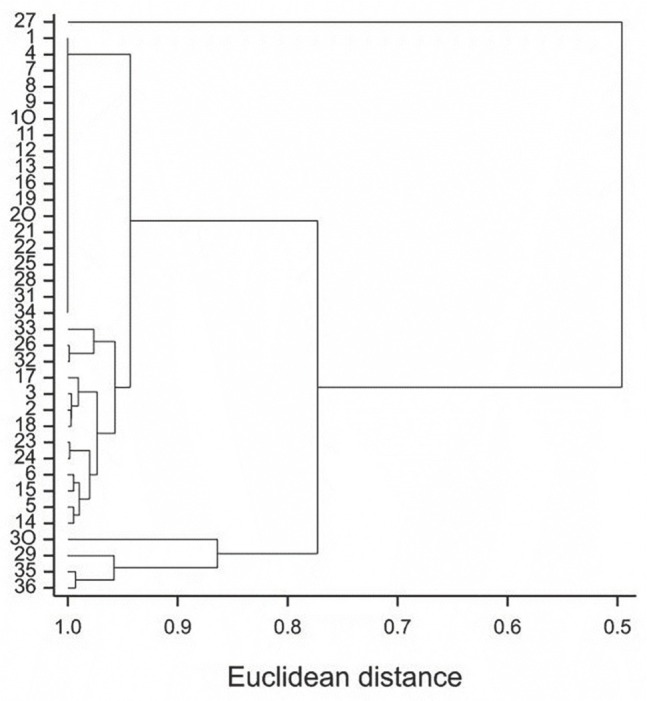
Dendrogram of the nearest neighbour cluster grouping of combinations of temperature, cultivars, and variants on the basis of four traits.

In the presented dendrogram, as a result of the nearest neighbour grouping using the Euclidean distances method, all the examined combinations were divided into three groups Fig. 1. The first group comprised four combinations (29, 30, 35 and 36), and the second group contained only one combination (27).

**Figure 2:**
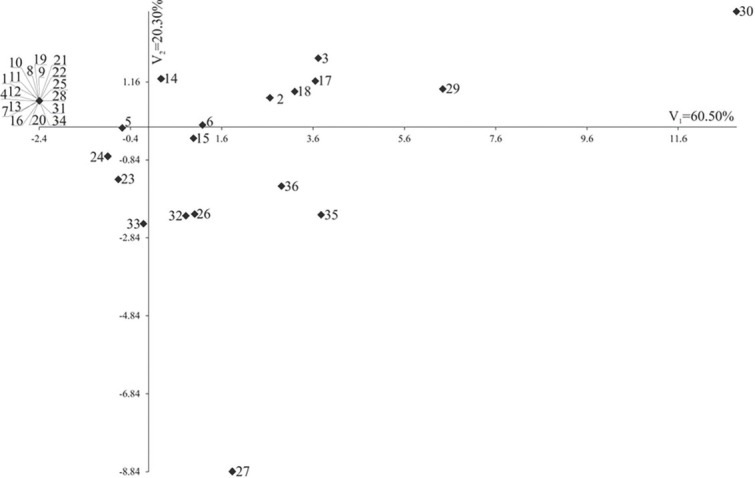
Distribution of 36 combinations of temperatures, cultivars, and variants in the space of the first two canonical variables.

Individual traits are of different importance and have a different share in the joint multivariate variation. A study on the multivariate variation for combinations also includes identification of the most important traits in the multivariate variation of combinations. Analysis of canonical variables is a statistical tool that makes it possible to solve the problem of multivariate relationships Seidler-Łożykowska et al., 2013; Lahuta et al., 2018; Wrońska-Pilarek et al., 2018; Bocianowski and Majchrzak, 2019. Figure 2 shows the variability of the four traits of 36 studied combinations of cultivars, temperatures, and variants in terms of the first two canonical variables. In the graph, the coordinates of the point for particular combinations are the values for the first and second canonical variables, respectively. The first two canonical variables accounted for 80.8% of the total multivariate variability between the individual combinations Fig. 2. The most significant, positive, linear relationship between the first canonical variables was found for immobile J2 after 24 h *r*=0.9737, *P*<0.001) and immobile J2 after 48 h *r*=0.8736, *P*<0.001). The second canonical variable was significantly negatively correlated with immobile J2 immersed in water *r*=-0.91, *P*<0.001) and immobile J2 after NaOH treatment *r*=-0.9102, *P*<0.001). The greatest variation in terms of all the traits jointly (measured Mahalanobis distances) was found for combinations 27 (21°C, Cultivar 1, J2+StS+water) and 30 (21°C, Cultivar 1, J2+StS+RD) (the Mahalanobis distance between them amounted to 16.379). The greatest similarity (0.00) was found for 18 combinations.

### Effect of *V. sativa* seeds on the expression levels of *hsp* genes in J2 stage

The known *hsp* gene sequences in *Caenorhabditis elegans* (Maupas, 1900) were the reference for the detection and identification of homologous *hsp* genes in *M. hapla*. Genome sequence and annotation have been imported from the WS246 release of WormBase Table 4. Sequences of the designed primers located on different exons of the *Mh*–*hsp*90, *Mh*–*hsp*1, *Mh*– *hsp*60, *Mh*–*hsp*43 and *Mh*–*hsp*12.3 genes are listed in Table 5.

**Table 4:** *Meloidogyne hapla* genes homologous to *Caenorhabditis elegans hsp* genes (based on WormBase version: WS246).

Hsp family	Gene *C. elegans* (Transcript ID)	Gene *M. hapla* (Transcript ID)	Gene location on contig
Hsp90	*hsp*90 (C47E8.5)	MhA1_Contig1972.frz3.gene3	4732..7425
Hsp70	*hsp*1 (F26D10.3)	MhA1_Contig113.frz3.gene45	79493..86067
Hsp60	*hsp*60 (Y22D7AL.5)	MhA1_Contig737.frz3.gene23	48523..50607
sHsps	*hsp*43 (C14F11.5)	MhA1_Contig30.frz3.gene18	50262..54876
sHsps	hsp12.3 (F38E11.1)	MhA1_Contig199.frz3.gene23	35708..36080

**Table 5:** List of primers used in the study.

Primer	Seq 5´ to 3´- Forward	Seq 5´ to 3´ - Reverse
*Mh-hsp*90	TCTCTGATGATGAGGCTGAAGA	TCACCGTCCTTCTTGTCCTT
*Mh-hsp*1	ACTCATCTTGGTGGTGAAGATT	TCAATGCCATCAAAGAGAGAATCA
*Mh-hsp*60	TTCCTGCTCTTGAATTGGCT	AATTGTGACTTCATCCGCCT
*Mh-hsp*43	CGTAGAGAAGAATTCCGTGAAGA	TTCAGAGCGGTGACTTCCA
*Mh-hsp*12.3	GCCTCTCCAGCATAATGACG	CGATTTATTTCACGACTGACTGA

Immersing the nematodes in the *V. sativa* seed diffusate (cv. Ina) resulted in increased expression of three *hsp* genes: *Mh*–*hsp*90, *Mh*–*hsp*1, and *Mh*–*hsp*43. The most significant changes occurred in the expression of the *Mh*–*hsp*43 gene. *Mh*– *hsp*43 gene expression was 2.6 compared to the control, 1.0. Lower expression occurred in the the gene *Mh*–*hsp*1 = 2.2 and gene *Mh*–*hsp*90 = 1.8. In the *Mh*–*hsp*60 (0.9) and *Mh*–*hsp*12.3 (0.2) genes, no increase in expression compared to the control was observed.

With the expression level increase of 1.8 and 1.7, respectively, the *Mh–hsp*43 gene and the *Mh–hsp*1 gene displayed the greatest difference in expression between the nematodes immersed in the diffusate (paralyzed) and those transferred from the diffusate to water (those nematodes regained their ability to move).

**Figure 3:**
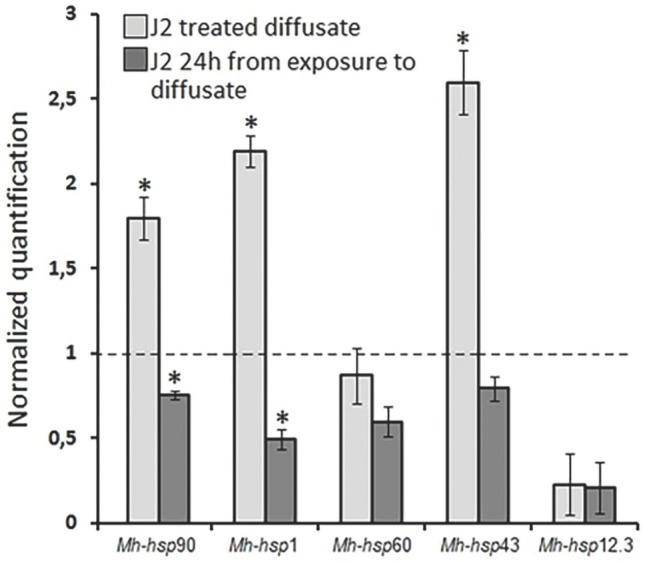
Influence of *Vicia sativa* cv. Ina diffusate treatment on *hsp* gene expression in *Meloidogyne hapla* J2 stage. Each value represents the mean ± s.d. of three biological replicates. The expression levels are indicated as the fold-change normalized to the control (untreated diffusate), normalized to the value of 1 (dashed line). Values were expressed as the mean fold difference, and statistically significant differences between treated and control samples are shown; *p≤0.01 based on *t*-Student test.

The expression level of tested *Mh*–*hsp*90 (0.8), *Mh*–*hsp*1 (0.5), *Mh*–*hsp*60 (0.6), *Mh*–*hsp*43 (0.8), and *Mh*–*hsp*12.3 (0.2) genes in J2 stage that regained the ability to move was lower than the control (1.0) Fig. 3.

## Discussion

The results of observation of the motility and mortality of J2 stage of the northern root–knot nematode, as well the expression profile of selected *hsp* genes, showed that common vetch seeds affect the J2 stage of *M. hapla*, which is important for the population dynamics of this root-knot nematode species.

When exposed to diffusates of common vetch seeds, invasive J2 were found to lose their motility, which was probably due to the occurrence of oxidative stress caused by the action of HCN present in the diffusate. It has been proven that HCN is toxic to nematodes, and at a relevant concentration, it causes paralysis and even death of specimens of the model organism, the free-living nematode *Caenorhabdidtis elegans* Gallagher and Manoil, 2001; Curto et al., 2012; Ayuda-Durán et al., 2020. The motile behaviour of nematodes determines their ability as a parasitic species to invade the root tissues of the host plant and their further development within Gray and Lissmann, 1964. The paralysis and mortality of the invasive J2 stages immersed in common vetch seed diffusates indicate that, even at the stage of seeds in the soil, plants may have a limiting effect on invasive nematodes that are potentially able to infect them. The presence of cyanogenic compounds and the release of HCN into the soil may explain the slight increase in the nematode population observed in the natural environment where *V. sativa* Dobosz and Krawczyk, 2019; Mosjidis et al., 1994 and other species of the genus *Vicia* are grown (Moneim and Bellar, 1993). Similarly, the poor suitability of *Sorghum* spp. and its inter-specific hybrids as plant hosts of *M. incognita* (Kofoid & White, 1919) Chitwood 1949 and *M. chitwoodi* Golden et al. (1980) Mojtahedi et al., 1993; Curto et al., 2012 is also attributable to the presence of cyanogenic compounds.

The increased expression of the *Mh*–*hsp*90, *Mh*–*hsp*1 (Hsp90 and Hsp70 protein family), and *Mh*–*hsp*43 (sHsp protein family) genes observed in J2 stage specimens was a component of the body’s response to environmental stress conditions such as common vetch (cv. Ina) seed diffusates. It was also observed that the increase in the level of expression of these genes was accompanied by the motility loss in J2 stage specimens. Our observations are consistent with previous literature reports regarding the chaperone role of the indicated *hsp*90 and *hsp*1 genes in the protection of cells against oxidative stress and their involvement in maintaining homeostasis Kegel et al., 1996; Wang et al., 2004; Haslbeck et al., 2005; Ungelenk et al., 2016; Balchin et al., 2016; Fernandez-Fernandez et al., 2017; Schopf et al., 2017; Craig, 2018; Nillegoda et al., 2018; Mok et al., 2018; Janowska et al., 2019; Doyle et al., 2019; Genest et al., 2019. Not only our study, but also other studies concerning three species of *Meloidogyne* nematodes: *M. artiellia*, *M. incognita* and *M. hapla*, have shown that changes in the environment of these nematodes also caused changes in the expression of *hsp*90 gene. A significant increase in expression of this gene was observed as a result of thermal (heat and cold) stress, heavy metals stress, and inorganic compounds Bai et al., 2014; De Luca et al., 2009; Wu et al., 2019a. The marked increase in the expression of the *hsp*90 gene as a result of the action of various environmental stressors implies that this gene can be used as a potential bioindicator of the environmental impact on the organism of Meloidogyne nematodes Bai et al., 2014. The *hsp*1 gene encodes the Hsp70 protein in the *C. elegans*, which is critically required during early juvenile stage in the development of this nematode Papsdorf et al., 2019. The research showed that overexpression of *hsp*1 leads to locomotion defects in *C. elegans* Papsdorf et al., 2014. Our research also shows an increase in expression of *Mh*–*hsp*1 gene and a decrease in motor activity in *M. hapla*. However, additional studies are needed to confirm this relationship. According to Bai et al. (2014), Hsp90 as a potential bioindicator should be applied simultaneously with Hsp70 to reflect the environmental influence on organisms more accurately.

The highest increase in expression as a result of exposure of the J2 stage of *M. hapla* to the common vetch seed diffusate of cv. Ina was observed in the *Mh*–*hsp*43 gene. The study shows an association between the increased expression of *Mh*–*hsp*43 gene and the adverse effect of common vetch seeds (cv. Ina) with a high content of cyanogenic compounds. These genes encode proteins belonging to the small heat shock proteins (sHsp). sHsp is a family of molecular chaperones characterized by a low molecular weight of 12–43 kDa. The primary function of sHsp is to maintain protein homeostasis in response to a variety of stress conditions, including heat shock, hyperosmosis, starvation, and oxidative stress Haslbeck et al., 2005; Janowska et al., 2019; Kegel et al., 1996; Ungelenk et al., 2016; Wang et al., 2004. Hsp43 expression was observed in all developmental stages of *C. elegans* (Ding and Candido, 2000). In *C. elegans*, the Hsp43 protein has been shown to be expressed in the epidermis and plays an important role in the resistance of this organism to heat stress (Liu et al., 2018; Fu et al., 2020). The tested expression of the *Mh-hsp*43 gene was 1.6 times higher than in the control sample, which indicates a significant role of this gene in the response to oxidative stress caused by the exposure of the J2 stage of *M. hapla* to the diffusate from *V. sativa* seeds cv. Ina.

Unlike with the *Mh*–*hsp*90, *Mh*–*hsp*1, and *Mh*–*hsp*43 genes, we did not observe an increase in the expression level of the *Mh*–*hsp*60 and *Mh*– *hsp*12.3 genes in the study. The *Mh*–*hsp*60 gene is responsible for encoding Hsp60 protein, which is probably involved in the protection of the mitochondria against the negative effects of heat stress Ewalt et al., 1997; Xu et al., 2010 and is indicated as a potential biomarker of toxic stress in nematodes Kammenga et al., 1998. The increase in Hsp60 protein synthesis in nematode *Plectus acuminatus* Bastian 1865 treated with copper or cadmium, as well as unaltered expression levels of *Mh*–*hsp*60 in J2 of *M. hapla* treated with common vetch seed diffusates, suggests that *Mh*–*hsp*60 gene is not involved in nematode stress response to HCN. We conjecture that the concentration of HCN in the environment was too low or the exposure time was not long enough to induce a marked increase in the expression of *Mh*–*hsp*60 gene. It can be assumed that the absence of an increase in the expression level of *Mh*–*hsp*12.3 gene may also be down to the same factors as mentioned for *Mh*–*hsp*60 gene.

Our study has demonstrated the negative impact exerted by common vetch seeds on the motility of the northern root-knot nematode J2 stage. The behaviour of the J2 stage, characterized by the loss of motility in these nematodes, was accompanied by an increase in the expression of the stress genes *Mh*–*hsp*90, *Mh*–*hsp*1, and *Mh*–*hsp*43. The *hsp*90 gene could be a potential bioindicator of the effects of the environment on the *Meloidogyne* organism. The admixture of common vetch seeds to arable and horticultural soils could become an effective method of limiting *M. hapla* population growth and its damage potential to crops. However, apart from being highly effective, it should be proven to not adversely affect the crop itself and, last but not least, to not pose a risk to food safety. Introducing common vetch seed as a soil additive may contribute to reducing weed growth as well as limiting nematode development in weed roots.
